# Comparison Study of Metabolic Syndrome's Differences and Diagnostic Criteria's Applicability among Xingjiang Uighur, Kazak and Han Population

**DOI:** 10.1155/2012/212383

**Published:** 2012-09-10

**Authors:** Sheng Jiang, Zijing Xie

**Affiliations:** Department of Endocrinology, The First Affiliated Hospital of Xinjiang Medical University, Urumqi 830013, China

## Abstract

Our paper is a study about metabolism syndrome (MS) incidence situations of different nationalities, including Uighur, Kazak and Han nationality in Xinjiang by means of a cross-sectional survey and compare differences and adaptabilities of applications of the diagnostic criteria for MS recommended by Adult Treatment Protocol III of National Cholesterol Education Program of America (ATP III), International Diabetes Federation (IDF) and Chinese Diabetes Society (CDS) in three groups of populations. Conclusion tell us, for Uighur population and Kazak population, IDF criterion and ATPIII criterion had a better consistence, and CDS criterion was worst. For Han population, CDS criterion and IDF criterion had a better consistence, and ATPIII criterion was worst. For the screening of MS incidence rate of Uighur and Kazak adult populations in Xinjiang region, ATPIII criterion was optimal, while CDS criterion was optimal for Han population. However, as for screening of clustering of multiple risk factors of MS, IDF criterion was better than other criteria for the three nationalities.

## 1. Introduction

Metabolism syndrome (MS) mainly manifests the clustering of hypertension (HP), obesity, lipid abnormality, hyperglycemia, and insulin resistance (IR) in individuals and rapid increase of its incidence rate has become a global public health focus. However, the adaptabilities of different diagnostic criteria in different populations are always debatable [1]. According to the incidence rate indicated by MS related epidemiological data, it is well known that MS incidence rate is apparently different form different races and nationalities. For MS incidence rate in Xinjiang region, China, Uighur (Uighur nationality) is 35.2%, Han nationality is 9.21%, and Kazak (Kazak nationality) is 20.1%. There are significant differences [2]. Lu et al. reported that when MS was diagnosed for different nationalities of residents in Xinjiang region, there are differences for diagnosis tangency of waist circumference (WC), and it was different from the research result of inland Han population [3]. 

At present, there are at least 8 diagnostic criteria of MS in the world [4]. As various criteria themselves have some defects, even if differently defined criteria are used in the same population, it is obvious that different detection rates will be obtained. However, as different nationalities and races have different MS incidence rates, at present there is no still no unified judgment criterion for MS internationally [5]. Therefore, the differences for total incidence rate of MS detected by different diagnostic criteria are objectively existent among the same and different populations. For the detection rate of all components of MS among population detected by different criteria, such as obesity, hyperglycaemia, lipid abnormality, HP and IR, the differences of the results are greater. The main reasons for these differences lie in that various diagnostic criteria adopt different measurement indicators, and they highlight different parts for effects of 4 indicators in MS judgment. Also, used measurement methods and dividing values are also inconsistent [6], which causes chaos in this field to a certain extent. However, it is very difficult to prepare a globally unified criterion mainly because that there are apparent differences between races and between regions. Therefore, it is the most important in fact to prepare the most suitable criteria for different races and nationalities. 

This study respectively adopted the diagnostic criteria of MS recommended by International Diabetes Federation (IDF), Adult Treatment Protocol III National Cholesterol Education Program of America (ATP III), and Chinese Diabetes Society (CDS) to analyze MS of Uighur, Kazak and Han populations in Xinjiang and incidence situations of various components and compare the consistence of 3 criteria diagnosing MS of 3 groups of populations in order to further understand the epidemiological situations of MS of Uighur, Kazak and Han populations in Xinjiang, investigate screening consistence and population suitability of currently used 3 diagnostic criteria and provide a basis for further conducting targeted health education and clinical intervention and provide the necessary based evidence for subsequently revising or preparing diagnostic criteria of MS for different nationalities. 

## 2. Objects and Methods

### 2.1. Objects

A cluster-sampling survey was carried out for Uighur, Kazak, and Han residents from 12 units in Urumqi city, Xinjiang, a multistage-cluster sampling survey was carried out for in Uighur, Kazak and Han residents in Aletai city and Kashgar city and their surrounding countries and towns. There were a total of 6928 persons aged 30 to 80 years old. Although 7140 persons should have been surveyed, 6928 persons were investigated in fact. The response rate was 97.03%. Among them, there were 2053 Uighur persons (male: 969; female: 1084), 2219 Kazak persons (male: 1016; female: 1203) and 2656 Han persons (male: 1588; female 1068). This study was conducted in accordance with the declaration of Helsinki. This study was conducted with approval from the Ethics Committee of the First Affiliated Hospital of Xinjiang Medical University. Written informed consent was obtained from all participants.

### 2.2. Methods

Demographic indicators and health questionnaire included age, gender, diabetes mellitus history, hypertension history, and so forth. The questions were asked by professionally trained investigators. According to the methods, blood pressure, height, body weight and waist circumference (WC) were measured, and body mass index (BMI) was calculated. Laboratory test indicators included fasting blood glucose (FPG), total cholesterol (TC), triglyceride (TG), high-density lipoprotein cholesterol (HDL-C), low-density lipoprotein cholesterol (LDL-C), and blood glucose at 2 h of OGTT test (PPG). 

### 2.3. Diagnostic Criteria of MS (Table 1)

ATP III reversion criterion [7] complies with the following 3 items or more: (1) obesity: WC ≥90 cm (male foreign citizen of Asian origin) or ≥80 cm (female foreign citizen of Asian origin); (2) high TG: namely TG ≥1.70 mmol/L; (3) low HDL-C: HDL-C <1.04 mmol/L (male) or <1.30 mmol/L (female); (4) dysarteriotony: systolic pressure (SBP)/diastolic pressure (DBP) ≥130/85 mmHg; (5) FPG abnormality: FPG ≥5.6 mmol/L. IDF criterion: comply with (1) central obesity and two items from (2) to (5) or more: (1) central obesity: WC ≥90 cm (male Chinese) or ≥80 cm (female Chinese); (2) high TG: TG ≥1.70 mmol/L or the cases with diagnosed high TG albumosemia receiving treatment; (3) low HDL-C: HDL-C <1.04 mmol/L (male) or <1.30 mmol/L (female), or the cases with diagnosed low HDL-C albumosemia receiving treatment; (4) dysarteriotony: SBP/DBP ≥130/85 mmHg, or the cases with diagnosed hypertension receiving treatment; (5) FPG abnormality: FPG ≥5.6 mmol/L or the cases with with diagnosed type II diabetes mellitus. CDS criterion [8]: comply with 3 items of the following 4 components or all: (1) overweight and (or) obesity: BMI ≥0.25  (kg/m^2^); (2) hyperglycaemia: FPG ≥6.1 mmol/L and PPG ≥7.8 mmol/L, and (or) the cases with diagnosed diabetes mellitus receiving treatment; (3) hypertension: SBP/DBP ≥140/90 mmHg, and (or) the cases with diagnosed hypertension receiving treatment; (4) lipids disorder: TG ≥1.7 mmol/L, and (or) HDL-C <0.9 mmol/L (male) or <1.0 mmol/L (female). 

### 2.4. Statistical Analysis

Measurement data were expressed as $\overset{-}{x}\pm s$, and enumeration data were expressed as rate and constituent ratio. “Xinjiang Statistics Yearbook” 2006 Edition and Population Statistics Data in Xinjiang in 2005 were respectively taken as the standards to calculate the standardized incidence rate. According to calculated consistence rate, Youden'sindex and Kappa value, the consistence between two of three diagnostic criteria of MS was analyzed. In addition, SPSS17.0 statistics software was used for data analysis. 

## 3. Results

### 3.1. General Characteristics and Biochemical Results of ****Surveyed Uighur, Kazak, and Han Populations Were Shown in Table 2

#### 3.1.1. Comparisons of Detection Rates of Various Risk Factors of Uighur, Kazak, and Han Populations under 3 Diagnostic Criteria

For Uighur population, central obesity ratios of diagnosed MS cases according to ATP III, IDF, and CDS criteria were, respectively, 91.43%, 100%, and 91.58%, the hypertension ratios were, respectively, 40.95%, 50.06%, and 59.75%, high TG albumosemia ratios were respectively 86.67%, 81.58%, and 85.63%, low HDL-C albumosemia ratios were, respectively, 72.79%, 62.67% and 50.51%, and the ratios of cases with blood glucose increase were respectively 62.72%, 55.01%, and 72.69%. For cases with MS diagnosed by 3 criteria, the ratios of cases with multiple (≥3 factors) risk factors clustering were respectively 98.64%, 97.9% and 92.81%. For Kazak population, central obesity ratios of diagnosed MS cases according to ATP III, IDF and CDS criteria were respectively 98.83%, 100% and 92.57%, hypertension ratios were respectively 93.79%, 90.94% and 91.34%, high TG albumosemia ratios were respectively 29.32%, 28.30% and 33.66%, low HDL-C albumosemia ratios were respectively 50.68%, 56.79% and 32.67%, and the ratios of cases with blood glucose increase were respectively 46.80%, 45.66% and 69.80%. For cases with MS diagnosed by 3 criteria, the ratios of cases with multiple (≥3 factors) risk factors clustering were respectively 100.00%, 96.23% and 91.34%. For Han population, central obesity ratios of diagnosed MS cases according to ATP III, IDF and CDS criteria were respectively nationality 81.20%, 100% and 72.77%, the hypertension ratios were respectively 66.30%, 62.86% and 36.15%, high TG albumosemia ratios were respectively 67.41%, 61.85% and 76.06%, low HDL-C albumosemia ratios were respectively 52.79%, 50.25% and 41.78%, the ratios of cases with blood glucose increase were respectively 78.13%, 74.79% and 88.73%. In addition, the ratios of cases with multiple (≥3 factors) risk factors clustering were respectively 100%, 98.49% and 89.91% (Table 3). 

### 3.2. Incidence Rates of Uighur, Kazak and Han Populations under the Three Diagnostic Criteria of MS

For Uighur population, incidence rates (standardized incidence rates) of cases with MS respectively diagnosed according to ATP III, IDF and CDS criteria were respectively 35.80% (29.64%), 39.41% (35.88%) and 23.72% (19.17%). The detection rate for IDF criterion was highest, and detection rate of MS for CDS criterion was lowest. For Kazak population, incidence rates (Standardized incidence rate) of cases with MS respectively diagnosed according to ATP III, IDF and CDS criteria were respectively 23.21% (21.29%), 23.88% (21.96%) and 18.21% (16.65%). The detection rate of MS for IDF criterion was highest, and the detection rate of MS for CDS criterion was lowest. For Han population, incidence rates (standardized incidence rate) of cases with MS respectively diagnosed according to ATP III, IDF and CDS criteria were respectively 27.03% (27.18%), 22.40% (20.39%) and 16.04% (16.02%). The detection rate of MS for ATP III criterion was highest, and the detection rate for MS for CDS criterion was lowest. See Table 4. 

### 3.3. Comparisons of Consistence of MS Incidence Rate as Uighur, Kazak and Han Populations Were Screened by the Three Criteria

For Uighur population, diagnosis consistence rate, Youden's index and Kappa value between ATPIII criterion and IDF criterion were highest (0.9026, 0.7801 and 0.7928). For Kazak population, diagnosis consistence rate, Youden's index and Kappa value between ATP III criterion and IDF criterion were highest (0.9878, 0.9568 and 0.9662). For Han population, diagnosis consistence rate, Youden's index and Kappa value between CDS criterion and IDF criterion were highest (0.7172, 0.1106 and 0.0953) (Table 5). 

## 4. Discussion 

MS is the clustering status of a group of risk factors closely related to cardiovascular disease, and obesity or overweight, blood glucose increase, lipids disorder and HP are its main components. Its incidence rate rapidly increases in recent 20 years, and it has become a hot issue of common concern at home and abroad in recent years [9, 10]. Also, MS is a clinical syndrome and a clustering status of multiple metabolism risk factors of cardiovascular and cerebrovascular diseases in the same body (metabolic disorder-related risk factors of cardiovascular and cerebrovascular disease) [11]. At present, MS has become a novel chronic disease and a public health problem. MS is an important risk factor causing increases of incidence rate and mortality rate of diabetes mellitus and cardiovascular disease. With caloric intake increase, physical activity decrease and obesity prevalence of global human, MS incidence rate will still rises [12, 13]. Clinical studies show the incidence risk of chronic diseases of MS patients such as cardiovascular disease (CVD), diabetes mellitus and hypertension, is farther higher than that of normal people [14–16]. Therefore, it is very significant to timely and accurately judge MS and timely conduct intervention in clinic [17]. However, diagnostic criteria of MS and adaptabilities of diagnostic criteria in different regions and different populations have great differences and arguments. So, there is always no unified diagnostic criterion of MS [18]. Currently, more common diagnostic criteria in China are ATP III criterion, CDS criterion and IDF criterion. Xinjiang is the accumulation area of multiple nationalities, and Uighur nationality, Kazak nationality and Han nationality are main nationalities. Many clinical studies suggest that incidence rates of many chronic diseases such as hypertension, diabetes mellitus and MS are different among Uighur, Kazak and Han populations in Xinjiang region [19], which is possibly associated with the dietary habits, life styles and genetic factors of various nationalities and their interactions [20]. 

The study respectively used three common diagnostic criteria of MS, namely ATP III, IDF and CDS to carry out screening for the same population among Uighur, Kazak and Han populations. As a result, it was found that for Uighur nationality and Han nationality, MS incidence rates (standardized incidence rate) for ATP III criterion were highest, respectively 35.80% (29.64%) and 27.03% (27.18%), and both MS detection rates for CDS criterion were lowest; For Kazak population, the detection rate for IDF criterion was highest 23.88% (21.96%), and MS detection rate of CDS criterion was lowest. As for single-risk factor, detection rates for three criteria had great differences, which was related to different emphases of various criteria; For screening of MS risk factor clustering, ATP III criterion was most sensitive for Uighur, Kazak, and Han populations (98.64%, 100% and 100%), and the detection rate for CDS criterion was lowest (92.81%, 91.34%, and 89.91%), indicating that as MS-related studies and prevent and treatment were carried out for Uighur, Kazak, and Han residents in Xinjiang region, the detection rate for ATP III criterion was highest for Uighur nationality and Han nationality, and the detection rate for IDF criterion was highest for Kazak nationality. However, for screening of risk-factor clustering, ATP III criterion was better than the other criteria for the three populations. 

Comparisons of consistence of MS incidence rate of Uighur, Kazak, and Han populations diagnosed by the three criteria showed that for Uighur nationality and Kazak nationality, consistence rate, Youden's index and Kappa value between ATP III criterion and IDF criterion were highest, indicating that for Uighur and Kazak populations, ATP III criterion and IDF criterion had a better consistence, and the consistence rate between CDS criterion and the other two criteria (ATP III and IDF) wasn't high. For Han population, consistence rate, Kappa value, and Youden's index between CDS criterion and IDF criterion were highest, while consistence rate, Kappa value, and Youden's index between ATP III and IDF criterion were lowest. It is suggested that ATP III criterion and IDF criterion are relatively more suitable for Uighur and Kazak populations, rather than Han population. For Han population in Xinjiang region, CDS criterion adaptability was best, IDF criterion is second, and ATP III is the most unsuitable. 

According to the analysis of this study, it is found that MS definition itself has some defects. For the judgment and treatment of risk factors of high-risk population, it is necessary to select the most suitable criterion for this population on the base of comprehensive consideration of existent generally-accepted diagnostic criteria and treatment principle. For Uighur, Kazak, and Han populations in Xinjiang region, diagnostic criteria of MS shall be different due to their different genetic factors and life styles and differences for physical characteristics, and body metabolism. The study suggests that ATP III criterion is preferred for screening of MS incidence rate of adult Uighur and Kazak populations, while CDS criterion ispreferred for Han population. For screening of clustering of MS multiple-risk factors, IDF criterion is better than the other criteria for the three nationalities. 

## Figures and Tables

**Table 1:** Comparison of three diagnostic criterions for metabolic syndrome.

Item	ATP III*	IDF^Δ^	CDS^#^
Obesity or central obesity	WC ≥90 cm (male) or ≥80 cm (female)	WC ≥90 cm (male) or ≥80 cm (female)	BMI ≥0.25 (kg/m^2^)
High TG	TG ≥1.70 mmol/L	TG ≥1.70 mmol/L or diagnosed as high TG-hematic disease and received therapy	TG ≥1.7 mmol/L
Low HDL-C	HDL-C <1.04 mmol/L (male) or <1.30 mmol/L (female)	HDL-C <1.04 mmol/L (male) or <1.30 mmol/L (female), or diagnosed as low-HDL-C hematic disease and received therapy	And (or) HDL-C <0.9 mmol/L (male) or <1.0 mmol/L (female)
Hypertension	SBP/DBP ≥130/85 mmHg	SBP/DBP ≥130/85 mmHg, or diagnosed as hypertension and received therapy	SBP/DBP ≥140/90 mmHg, and (or) diagnosed as hypertension and received therapy
Glucose/Lipid metabolic abnormal		FPG ≥5.6 mmol/L or diagnosed as type 2 diabetes	FPG ≥6.1 mmol/L and /PPG ≥7.8 mmol/L, and (or) diagnosed as type 2 diabetes

*Comply with at least any three of the five items; ^Δ^Any two items plus obesity or central obesity; ^#^possess three of the four items or all (High TG and low HDL-C was considered as one item).

**Table 2:** Clinical and biochemistry data of Uighur, Kazak, and Han population ($\bar{x}\pm s$).

Item	Uighur population		Kazak population		Han population	
	Male	Female	Male	Female	Male	Female
Age (year)	49.44 ± 13.07	46.43 ± 11.39	51.85 ± 12.23	43.24 ± 8.17	48.42 ± 12.14	44.91 ± 10.58
BMI (Kg/M^2^)	25.63 ± 4.29	26.34 ± 4.8	29.07 ± 4.42	27.87 ± 5.02	23.96 ± 4.39	26.07 ± 4.92
FPG (mmol/L)	5.58 ± 2.59	5.32 ± 1.9	4.99 ± 1.04	4.85 ± 1.00	5.76 ± 1.40	5.46 ± 0.99
PPG (mmol/L)	7.1 ± 3.8	6.87 ± 3.21	7.64 ± 1.92	7.60 ± 1.95	7.19 ± 3.34	5.84 ± 2.03
SBP (mmHg)	116.9 ± 17.9	114.6 ± 19.4	133.44 ± 25.81	130.18 ± 25.36	121.40 ± 20.11	112.86 ± 15.50
DBP(mmHg)	74.5 ± 11.8	74.1 ± 12.5	86.04 ± 15.34	83.17 ± 15.33	76.76 ± 11.58	73.89 ± 10.18
TC (mmol/L)	3.29 ± 1.49	3.38 ± 1.49	4.92 ± 0.88	4.72 ± 0.90	5.26 ± 1.01	5.03 ± 1.04
TG (mmol/L)	2.89 ± 1.95	2.61 ± 1.73	1.13 ± 0.98	1.91 ± 0.69	1.53 ± 0.95	1.41 ± 1.05
HDL-C (mmol/L)	1.22 ± 0.32	1.23 ± 0.31	1.55 ± 0.52	1.49 ± 0.45	1.50 ± 0.55	1.44 ± 0.55
LDL-C (mmol/L)	1.49 ± 1.60	1.63 ± 1.64	2.08 ± 1.15	1.89 ± 0.98	2.84 ± 1.03	2.60 ± 1.08
WC (cm)	89.86 ± 12.12	89.23 ± 12.51	95.18 ± 11.61	90.26 ± 12.41	84.61 ± 10.11	80.32 ± 10.51

**Table 3:** MS's risk factors detectable rate of Uighur, Kazak, and Han population diagnosed by three criterions (%).

MS five components	Uighur population			Kazak population			Han population		
	ATP III (%)	IDF (%)	CDS (%)	ATP III (%)	IDF (%)	CDS (%)	ATP III (%)	IDF (%)	CDS (%)
Central obesity (abdominal girth ≥90/80 cm)	91.43	100	91.58	98.83	100.00	92.57	81.20	100	72.77
High TG (TG ≥1.7 mmol/L)	86.67	81.58	85.63	29.32	28.30	33.66	67.41	61.85	76.06
Low HDL-C (<1.04/1.3 mmol/L)	72.79	62.67	50.51	50.68	56.79	32.67	52.79	50.25	41.78
BP abnormal (≥130/85 mmHg)	40.95	50.06	59.75	93.79	90.94	91.34	66.30	62.86	36.15
BG abnormal (FPG/PPG ≥5.6/7.8 mmol/L)	62.72	55.01	72.69	46.80	45.66	69.80	78.13	74.79	88.73
≥3 risk factors together	98.64	97.9	92.81	100.00	96.23	91.34	100.00	98.49	89.91

**Table 4:** MS's morbidity of Uighur, Kazak, and Han population diagnosed by three criterions among different age groups (%).

Age (years)	Uighur population			Kazak population			Han population		
	ATP III (%)	IDF (%)	CDS (%)	ATP III	IDF (%)	CDS (%)	ATP III	IDF (%)	CDS (%)
30~	27.58	28.71	13.86	14.06	14.85	9.79	28.40	23.83	14.69
40~	38.65	43.43	22.91	18.44	19.60	11.96	25.29	23.12	16.60
50~	43.83	48.29	34.38	39.85	40.23	32.18	28.78	21.43	15.31
60~	43.02	47.09	33.72	25.58	25.58	26.25	26.36	20.92	16.74
70~	25.82	35.00	22.50	18.84	18.84	17.39	24.21	16.84	23.16

Total	35.80	39.41	23.72	23.21	23.88	18.21	27.03	22.40	16.04

Standardized rate	29.64	35.88	19.17	21.29	21.96	16.65	27.18	20.39	16.02

**Table 5:** Comparison of diagnosis consistency of three criterions among Uighur, Kazak, and Han population.

Diagnostic criteria	Uighur population			Kazak population			Han population		
	Coincidence rate	Youden index	Kappa value	Coincidence rate	Youden index	Kappa value	Coincidence rate	Youden index	Kappa value
ATP III & IDF	0.9026	0.7801	0.7928	0.9878	0.9568	0.9662	0.6269	0.0003	0.0003
ATP III & CDS	0.756	0.5354	0.4572	0.8526	0.6120	0.5530	0.6687	0.0471	0.0369
IDF III & CDS	0.7837	0.6301	0.5132	0.8513	0.6219	0.5547	0.7172	0.1106	0.0953
